# LncRNA ZFAS1 in hepatocellular carcinoma: A systematic review of molecular mechanisms and clinical translation

**DOI:** 10.1016/j.ncrna.2025.11.003

**Published:** 2025-12-05

**Authors:** Peng Zhu, Hui-ying Liu

**Affiliations:** a Department of Hepatic Surgery (III), The Third Affiliated Hospital of Naval Medical University, Shanghai, 200433, China; b Department of Biotherapy, The Third Affiliated Hospital of Naval Medical University, Shanghai, 200433, China

**Keywords:** Hepatocellular carcinoma, ZFAS1, Long non-coding RNA, Competing endogenous RNA, Tumor microenvironment, Multidrug resistance

## Abstract

Hepatocellular carcinoma (HCC) remains a leading cause of cancer-related mortality globally, with its high recurrence rate and therapeutic resistance underscoring the urgent need for breakthrough molecular targets. The long non-coding RNA ZFAS1 has emerged as a critical regulatory hub in HCC pathogenesis through its multidimensional mechanisms. Clinical investigations reveal significant ZFAS1 overexpression in HCC tissues, which is strongly associated with microvascular invasion, lymph node metastasis, and unfavorable clinical outcomes. Meta-analytical data further corroborate its independent prognostic value in survival prediction.

Mechanistically, ZFAS1 functions as a competitive endogenous RNA (ceRNA) that sequesters tumor-suppressive miRNAs including miR-150 and miR-193a-3p, thereby de-repressing downstream oncogenic targets such as ZEB1/MMP14 and RALY/HGF/c-Met. This molecular interplay drives epithelial-mesenchymal transition (EMT) and metastatic progression, while ZFAS1-encoded micropeptides concurrently inhibit ferroptosis through mitochondrial ROS modulation and the miR-150/AIFM2 axis, thereby synergistically enhancing tumor proliferation and apoptotic resistance. Within the tumor microenvironment (TME), exosome-derived ZFAS1 remodels intercellular communication networks, promoting angiogenesis via STAT3/VEGFA signaling, though its immunometabolic regulatory mechanisms warrant further elucidation. Clinically, plasma ZFAS1 demonstrates enhanced diagnostic utility when combined with alpha-fetoprotein (AUC = 0.891), while therapeutic targeting of ZFAS1-mediated PI3K-AKT and PERK/ATF4 pathways shows promise in overcoming sorafenib/donafenib resistance. Current translational challenges include ZFAS1 isoform heterogeneity, suboptimal liquid biopsy sensitivity, and dynamic TME interactions.

Future directions should employ multi-omics integration (spatial transcriptomics/single-cell sequencing) coupled with AI-driven network modeling to systematically decode ZFAS1's regulatory architecture, ultimately enabling precision theranostic strategies for HCC management.

## Abbreviation

HCC: Hepatocellular carcinoma
PLC: Primary liver cancer
OS: overall survival
PFS: progression-free survival
mRNAs: messenger RNAs
lncRNAs: long non-coding RNAs
EMT: epithelial-mesenchymal transition
ZFAS1: ZNFX1 antisense RNA 1
ZNFX1: Zinc finger NFX1-type containing 1
snoRNA: small nucleolar RNA
SDT: sonodynamic therapy
ceRNA: competitive endogenous RNA
RIP: RNA immunoprecipitation
MDK: midkine
AIFM2: apoptosis-inducing factor mitochondria-associated 2
TNBC: triple-negative breast cancer
ccRCC: Clear cell renal cell carcinoma
PDAC: Pancreatic ductal adenocarcinoma
HES-1: HES family basic helix-loop-helix transcription factor 1
NICD: Notch intracellular domain
TME: tumor microenvironment
mOS: median overall survival
ORR: objective response rate
uHCC: unresectable HCC
MDR: multidrug resistance
ER: endoplasmic reticulum
IC50: half-maximal inhibitory concentration

## 1 Introduction

This systematic review adopted core PRISMA principles for literature retrieval [1]. Through comprehensive manual searches of MEDLINE, Embase, Web of Knowledge, LILACS, SciELO, Cochrane, PubMed databases, Google Scholar, and forward/backward citations (database inception to Mar 28, 2025), we identified and synthesized all relevant evidence on ZFAS1 in HCC pathogenesis.

Primary liver cancer (PLC) represents a formidable global public health challenge, with its disease burden continuing to escalate. Recent epidemiological surveillance indicates that 2020 witnessed approximately 905,700 new PLC diagnoses and 830,200 related deaths worldwide, solidifying its status as the sixth most prevalent malignancy and third leading cause of cancer mortality [[2], [3], [4], [5], [6], [7]]. Histologically, hepatocellular carcinoma (HCC) dominates PLC cases (80–90 %), while cholangiocarcinoma, fibrolamellar carcinoma, and other rare subtypes constitute the remaining cases [8]. Despite therapeutic advances including surgical resection, radiofrequency ablation, and liver transplantation, postoperative recurrence rates persist alarmingly at 70–80 % within five years, profoundly compromising clinical outcomes [[9], [10], [11]].

This clinical reality underscores the central paradox in contemporary HCC research: the imperative to decipher molecular mechanisms underlying therapeutic resistance and metastatic propensity, thereby enabling the development of precision interventions to enhance treatment responsiveness and mitigate recurrence risks. Recent years have witnessed a paradigm shift in translational medicine, focusing on dual optimization of overall survival (OS) and progression-free survival (PFS) through integrative approaches that bridge fundamental discoveries with clinical implementation. This strategic orientation not only offers novel solutions to overcome therapeutic bottlenecks in HCC management but also establishes a robust framework for personalized diagnostic and therapeutic systems.

Conventional dogma posits that only 2–3 % of the human genome comprises protein-coding genes capable of transcribing into messenger RNAs (mRNAs) [12]. The early-mid 2000s witnessed transformative advances in transcriptome profiling, as global genomic initiatives sought to refine proteome characterization through comprehensive RNA sequencing. These investigations yielded paradigm-shifting discoveries that fundamentally challenged conventional understanding of genetic expression. Systematic analyses revealed pervasive transcription of long non-coding RNAs (lncRNAs) across eukaryotic genomes —evolutionarily conserved transcripts exhibiting negligible protein-coding potential [[13], [14], [15], [16]]. This revelation contradicted the central dogma of molecular biology, establishing non-coding transcriptional landscapes as essential components of cellular regulation. LncRNAs have emerged as pivotal epigenetic regulators in tumor biology, demonstrating multifaceted roles in oncogenesis, metastasis, and therapeutic resistance through dynamic modulation of gene expression networks spanning epigenetic, transcriptional, and post-transcriptional levels [[17], [18], [19], [20], [21], [22]].

In HCC, lncRNAs exhibit diverse functional roles that profoundly influence the pathogenesis and progression of HCC [23]. These multifaceted roles encompass, but are not limited to: (1) precise regulation of tumor cell proliferation-apoptosis equilibrium via targeting core cell cycle regulators (e.g. Cyclin D1/CDK4 complexes) and apoptosis-associated genes (e.g. Bcl-2 family members) [24,25]; (2) modulation of canonical oncogenic pathways such as Wnt/β-catenin signaling. LncRNA FOXD2-AS1 epigenetically silences the Wnt/β-catenin signaling pathway inhibitor DKK1, thereby activating this signaling pathway and promoting tumor progression [26]; (3) Of particular clinical significance, metastasis-associated lncRNAs (e.g. *HOTAIR*, *NEAT1*) orchestrate HCC dissemination through transcriptional activation of epithelial-mesenchymal transition (EMT) master regulators (*Snail*, *Twist*) and subsequent upregulation of mesenchymal markers (*N-cadherin*, *Vimentin*) [27,28]; (4) Viral-mediated epigenetic remodeling, exemplified by HBV/HCV-induced H3K27ac enrichment at promoter regions, drives NEAT1 overexpression. This lncRNA facilitates chemoradioresistance by activating pro-survival autophagy and DNA repair mechanisms, underscoring its central role in HCC microenvironment adaptation [29,30].

**Fig. 1:**
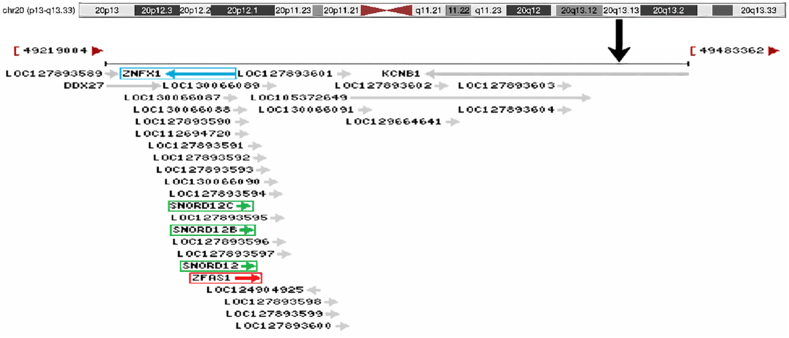
Schematic diagram of ZFAS1 gene structure. ZFAS1 (red) is located on the long arm of human chromosome 20 region 13.13 (20q13.13), transcribed from the antisense strand of the ZNFX1 gene (blue). It contains three small nucleolar RNA (snoRNA)—Snord12, Snord12B, and Snord12C (green).

ZFAS1 (ZNFX1 antisense RNA 1) is a long non-coding RNA transcribed antisense to the Zinc finger NFX1-type containing 1 (ZNFX1) gene locus on chromosome 20q13.13, harboring three small nucleolar RNAs (*SNORD12*, *SNORD12B*, *SNORD12C*) within its transcript structure [31] (Fig. 1). Initial functional characterization revealed that ZFAS1 silencing in mammary epithelial cells enhanced proliferative and differentiation capacities without affecting SNORD expression, while clinical data demonstrated its tumor-suppressive potential through significant downregulation in breast carcinoma specimens [32]. Contrastingly, accumulating evidence identifies ZFAS1 as a context-dependent oncogene, showing marked overexpression in HCC, gastric cancer, and colorectal cancer where it drives tumor progression via regulation of proliferation, angiogenesis, and metastatic dissemination [[33], [34], [35], [36], [37], [38]]. This molecular crosstalk exhibits both evolutionary conservation and tumor-type specificity. Collectively, ZFAS1 orchestrates multidimensional networks governing tumorigenesis, metastasis, and therapeutic resistance, positioning it as a promising target for liquid biopsy development and molecular therapeutics. Future investigations should employ multi-omics integration with clinical datasets to delineate microenvironment-modulated ZFAS1 regulatory circuits in HCC and other malignancies, ultimately advancing precision oncology applications.

While the oncogenic functions of ZFAS1 have been extensively characterized across multiple solid malignancies [[39], [40], [41], [42]], its pathophysiological significance in HCC remains incompletely delineated. This review systematically addresses this knowledge gap through five critical dimensions: (1) evaluating ZFAS1's clinical prognostic utility in HCC stratification, (2) elucidating its core regulatory networks driving malignant progression, (3) investigating pathway-specific interactions within HCC signaling cascades, (4) assessing its immunomodulatory roles in the tumor microenvironment, and (5) delineating resistance mechanisms to molecularly targeted therapies. Furthermore, we critically appraise ZFAS1's emerging potential as a liquid biopsy biomarker and propose innovative strategies to overcome current translational bottlenecks.

## 2 ZFAS1 expression profile characteristics in HCC and their clinical prognostic associations

ZFAS1 has emerged as a pivotal molecular driver in HCC, with its dysregulation demonstrating strong clinicopathological correlations. Li et al. [43] conducted a 113-patient cohort study revealing significant ZFAS1 upregulation in tumor tissues, showing robust associations with microvascular invasion (P = 0.028) and early postoperative recurrence (P = 0.041); Duan et al. [44] further validated these findings through TCGA database analysis (369 tumors vs 160 normal), where elevated ZFAS1 expression correlated with advanced clinical stage (P < 0.001) and lymph node metastasis (P = 0.001). Our multi-omics investigation [45] further established ZFAS1 overexpression patterns specifically associated with tumor progression markers including TNM staging and patient age (all P < 0.05). Survival analyses demonstrated significantly reduced OS in high-ZFAS1 patients (HR = 2.0), with multivariate Cox regression confirming its independent prognostic value (P < 0.01). These collective findings position ZFAS1 as a critical nexus connecting molecular pathogenesis with clinical manifestations through dual regulation of metastatic progression and therapeutic resistance. Given its dual utility as both diagnostic biomarker and therapeutic target, ZFAS1 represents a promising cornerstone for developing precision HCC management strategies.

The functional duality of ZFAS1 in HCC pathogenesis has generated significant academic debate. Wang et al. [46] reported tumor-suppressive characteristics, demonstrating ZFAS1 downregulation in HCC specimens correlated with poor prognosis (P < 0.05). Mechanistic dissection revealed ZFAS1-mediated demethylation of miR-9 promoter CpG islands, establishing a positive regulatory axis (r = 0.62) between these molecules. Furthermore, Shang et al. [47], observed ZFAS1 upregulation (P < 0.01) following sonodynamic therapy (SDT) with HMME-LND@C3F8-NBs, where elevated ZFAS1 expression exerted growth-inhibitory effects through undefined mechanisms. These paradoxical observations may originate from: (1) Cohort etiological heterogeneity (viral vs. non-viral HCC), (2) Technical variance in detection platforms (qPCR vs RNA sequencing), and (3) Microenvironment-dependent functional switching. Resolution requires multicenter validation integrating single-cell transcriptomics with CRISPR-Cas9-based spatial editing models to delineate ZFAS1's context-dependent regulatory logic in HCC progression.

Multiple meta-analyses have systematically validated ZFAS1's prognostic significance in HCC. In a meta-analysis encompassing 10 studies (n = 874 patients) across various solid tumors, including glioma, ovarian cancer, gastric cancer, colorectal cancer, non-small cell lung cancer, and HCC, Song et al. [48] demonstrated that elevated ZFAS1 expression was significantly associated with reduced overall survival (OS) (pooled HR = 1.58, 95 % CI: 1.28–1.97, P < 0.001) and increased risk of recurrence-free survival (RFS) events (HR = 1.90, 95 % CI: 1.29–2.79, P = 0.001). While this comprehensive analysis strongly supports ZFAS1 as a pan-cancer prognostic biomarker, specific subgroup findings for HCC within this study warrant careful interpretation. The HCC subgroup analysis (n = 113) indicated a trend towards increased RFS risk (HR = 1.76, 95 % CI: 1.05–2.94); however, the association with OS did not reach statistical significance (HR = 1.58, 95 % CI: 0.75–3.31), likely reflecting limited statistical power due to the relatively small HCC sample size within the meta-analysis. Lan et al. [49] (n = 841) confirmed ZFAS1's association with worsened OS (HR = 2.13, 95 % CI: 1.71–2.65) and RFS (HR = 2.00, 95 % CI: 1.45–2.77) (all P < 0.001), though without HCC-specific stratification. Liu et al. [50] (n = 978) similarly reported compromised OS (HR = 1.87, 95 % CI: 1.38–2.36, P < 0.001), with their HCC subgroup (n = 88) showing elevated risk (HR = 1.90, 95 % CI: 1.10–3.25), albeit requiring cautious interpretation due to sample constraints. Leng et al. [51] (n = 820) identified amplified OS risk in gastrointestinal malignancies containing HCC (HR = 2.03 vs overall HR = 1.97), suggesting tissue-specific microenvironmental modulation of ZFAS1's prognostic value. Notably, all studies employed fixed-effect models (I^2^ = 0 %) but shared limitations in fragmented HCC subgroup data. Future large-scale multicenter studies are imperative to develop HCC-specific prognostic frameworks.

To consolidate the clinical significance of ZFAS1 dysregulation in HCC, Table 1 systematically summarizes key studies elucidating its expression patterns, clinicopathological correlations, and prognostic utility across diverse cohorts.

**Table 1:** Summary of key clinical studies on ZFAS1 in HCC.

**Study (Year)**	**Sample Size**	**Methodology**	**Key Findings**	**Prognostic Value**
Li et al. (2015) [43]	113 patients	qPCR/IHC	ZFAS1↑ correlates with microvascular invasion (P = 0.028) and early recurrence (P = 0.041)	OS↓ (HR = 2.0, P < 0.01)
Duan et al. (2020) [44]	369 tumors vs 160 normals	Microarray and qRT-PCR	ZFAS1↑ associates with advanced stage (P < 0.001) and lymph node metastasis (P = 0.001)	Independent predictor
Zhu et al. (2023) [45]	150 patients	RNA-seq/Clinical correlation	ZFAS1↑ links to TNM stage and age (P < 0.05)	Diagnostic AUC = 0.87
Song et al. (2017) [48]	10 studies (n = 874)	Meta-analysis	Pooled OS↓ (HR = 1.58, 95 %CI:1.28–1.97, P < 0.001)	RFS risk↑ (HR = 1.90, P = 0.001)
Luo et al. (2018) [81]	60 HCC patients, 75 hepatitis B & cirrhosis, 79 controls	qRT-PCR	Plasma ZFAS1↑ in HCC vs controls (P < 0.001)	Diagnostic AUC = 0.801 (0.891 with AFP)

## 3 ZFAS1 orchestrates multifaceted regulatory networks in HCC malignant progression

ZFAS1 functions as a pivotal molecular hub in hepatocellular carcinoma (HCC), primarily through its role as a competitive endogenous RNA (ceRNA) to sponge tumor-suppressive microRNAs (miRNAs). Beyond this canonical mechanism, it also encodes functional micropeptides. This section synthesizes these multifaceted mechanisms into a coherent regulatory network, moving beyond a mere listing of interactions to provide a critical appraisal of how ZFAS1 integrates various oncogenic signals.

### 3.1 The central role of ZFAS1 as a competitive endogenous RNA (ceRNA)

The ceRNA activity of ZFAS1 constitutes its most characterized oncogenic function. We have consolidated these interactions not by the identity of the miRNA, but by the core biological outcomes they drive, revealing a convergent pathogenic strategy.

#### 3.1.1 ceRNA networks driving proliferation and survival

**Fig. 2:**
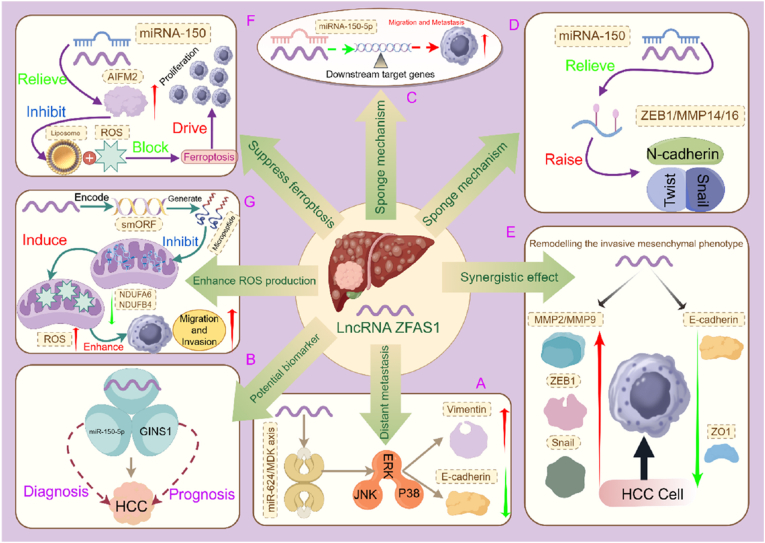
Multidimensional Regulatory Network of ZFAS1 in Hepatocellular Carcinoma. 2A. ZFAS1 accelerates hepatic/lung metastasis in vivo by activating the ERK/JNK/P38 signaling cascade via the miR-624/MDK axis, promoting Vimentin expression and suppressing E-cadherin. 2B. The ZFAS1/miR-150–5p/GINS1 regulatory axis serves as a potential biomarker for HCC diagnosis and prognosis (AUC >0.85, P < 0.01). 2C. Functional validation demonstrates that ZFAS1 promotes HCC cell proliferation, migration, and invasion by sponging miR-150–5p to relieve its suppression of downstream oncogenes. 2D. ZFAS1 promotes EMT and metastasis by sponging miR-150 to relieve transcriptional repression of the ZEB1/MMP14/16 complex, leading to upregulation of N-cadherin and EMT core transcription factors Twist/Snail. 2E. ZFAS1 overexpression drives the invasive mesenchymal phenotype in HCC cells by cooperatively activating MMP2/MMP9, ZEB1, and Snail, while suppressing epithelial junction proteins E-cadherin and ZO1. 2F. ZFAS1 competitively binds miR-150 to relieve its transcriptional repression of AIFM2, upregulating AIFM2 expression; as a key ferroptosis suppressor, AIFM2 blocks lipid peroxidation and ROS accumulation to inhibit ferroptosis and drive HCC cell proliferation. 2G. ZFAS1 encodes a functional micropeptide via a smORF that suppresses mitochondrial complex I subunits NDUFA6/NDUFB4, triggering ROS burst accumulation to enhance HCC cell migration and invasion. Created with Figdraw.

ZFAS1 robustly promotes HCC cell proliferation and tumor growth by simultaneously deregulating multiple cell cycle and survival pathways through distinct miRNAs. A key axis involves the sequestration of miR-624, which alleviates the repression of its target, the heparin-binding growth factor Midkine (MDK). Derepressed MDK activates downstream ERK/JNK/p38 MAPK signaling cascades, thereby driving cellular proliferation and suppressing apoptosis [44] (Fig. 2A). In a parallel and complementary pathway, ZFAS1 binds to miR-150–5p, leading to the upregulation of GINS1, a critical DNA replication initiator. This ZFAS1/miR-150–5p/GINS1 axis potently activates cell cycle progression and suppresses the p53 pathway, further fueling uncontrolled proliferation [52] (Fig. 2B). Further consolidating the pivotal role of the miR-150–5p interaction, our own functional validation [45] confirmed that ZFAS1 silencing suppresses HCC proliferation, migration, and invasion—effects that could be partially rescued by inhibiting miR-150–5p (Fig. 2C). Integrated pathway analysis of this axis specifically implicated the activation of both PI3K/Akt and JAK/STAT signaling networks, providing a mechanistic basis for its broad oncogenic effects. The convergence of these independent miRNA pathways (miR-624 and miR-150–5p) on pro-proliferative signaling highlights ZFAS1's role as a master coordinator of growth advantage in HCC.

#### 3.1.2 ceRNA networks orchestrating metastasis and EMT

The pro-metastatic function of ZFAS1 is largely mediated by its coordinated suppression of miRNAs that target master regulators of Epithelial-Mesenchymal Transition (EMT) and invasion. The most extensively studied interaction is with miR-150, whereby ZFAS1 sponging de-represses the expression of key EMT transcription factors like ZEB1 and SNAI1, as well as matrix metalloproteinases MMP14/16. This orchestrated program enhances extracellular matrix degradation and cellular motility, driving metastasis in vivo [39,43] (Fig. 2D,2 E). Furthermore, the conservation of this ceRNA logic is evident beyond HCC; in hepatoblastoma, ZFAS1 sequesters miR-193a-3p to activate the RALY/HGF/c-Met axis, a known driver of EMT and invasion [53]. This indicates that ZFAS1 employs a miRNA-guided "toolkit" to activate convergent pro-metastatic pathways across liver malignancies, albeit with tissue-specific miRNA-mRNA pairings.

#### 3.1.3 ceRNA networks in cell death and therapeutic resistance

Emerging evidence underscores ZFAS1's role in conferring resistance to cell death, a cornerstone of therapeutic failure. A seminal mechanism involves its sequestration of miR-150, which leads to the upregulation of Apoptosis-Inducing Factor Mitochondria-Associated 2 (AIFM2). AIFM2 is a known suppressor of ferroptosis, an iron-dependent form of regulated cell death. By enhancing AIFM2 expression, ZFAS1 protects HCC cells from lipid peroxidation and ROS accumulation, thereby promoting survival and conferring resistance to ferroptosis-inducing cues [54] (Fig. 2F). This mechanism provides a molecular link between ZFAS1 overexpression and the resilience of HCC cells, a theme expanded upon in Section 7 regarding drug resistance.

### 3.2 Non-canonical mechanisms: ZFAS1-Encoded micropeptides

Beyond its RNA-centric functions, ZFAS1 exemplifies the paradigm of lncRNAs encoding functional micropeptides. Specifically, ZFAS1 transcript contains small open reading frames (smORFs) that are translated into micropeptides. These micropeptides localize to mitochondria and directly interact with Complex I subunits (NDUFA6/NDUFB4), inducing a surge in mitochondrial reactive oxygen species (mtROS). This elevated mtROS state, contrary to inducing toxicity, paradoxically enhances the metastatic potential of HCC cells. This pro-metastatic effect is reversible upon treatment with ROS scavengers, providing functional validation [55] (Fig. 2G). This micropeptide-mediated mechanism operates independently of ZFAS1's ceRNA function, revealing an additional layer of regulatory complexity.

### 3.3 Synthesis and critical appraisal of ZFAS1 regulatory logic

The multidimensional networks orchestrated by ZFAS1 are synthetically cataloged in Table 2, which delineates miRNA targets, mechanistic consequences, and experimental validation approaches central to HCC pathogenesis.

**Table 2:** ZFAS1-Mediated miRNA Regulatory Axes in HCC Pathogenesis.

ZFAS1-driven Axis/Mechanism	Key Molecular Targets/Interactors	Functional Outcome in HCC	Experimental Validation
ZFAS1/miR-624/MDK	MDK, ERK/JNK/p38	Promotes proliferation, inhibits apoptosis	Luciferase, RIP, in vivo [44]
ZFAS1/miR-150–5p/Multiple Pathways	GINS1, PI3K/Akt, JAK/STAT	Drives cell cycle progression, proliferation, migration, and invasion	Dual-luciferase, Functional rescue, Pathway analysis [45,52]
ZFAS1/miR-150/EMT Master Regulators & MMPs	ZEB1, SNAI1, MMP2/9/14/16; (↓E-cadherin, ZO-1)	Induces full EMT program, enhances invasion & lung metastasis	RIP, RNA-pull down, luciferase, in vivo metastasis models [43]; Comprehensive review synthesizing EMT markers [39]
ZFAS1/miR-150/AIFM2	AIFM2	Inhibits ferroptosis, promotes survival	Luciferase, WB, Functional assays [54]
ZFAS1-encoded peptide/mtROS	NDUFA6, NDUFB4	Increases mitochondrial ROS, drives metastasis	Translation assays, Protein interaction, ROS detection [55]

A critical synthesis of the literature reveals that ZFAS1 functions as a central regulatory hub by deploying two primary strategies (1) as a ceRNA that sponges a repertoire of miRNAs (e.g., miR-150, miR-624, miR-193a-3p): to activate synergistic oncogenic pathways promoting proliferation, metastasis, and survival; and (2) as a source of micropeptides that directly modulate mitochondrial function to drive metastasis.

The recurring identification of miR-150 as a key partner across multiple studies [43,52,54] suggests it may be a predominant miRNA target of ZFAS1 in HCC. However, the reporting of other miRNAs like miR-624 and miR-193a-3p likely reflects context-dependent regulatory specificity and methodological biases in miRNA discovery (e.g., RIP-seq vs. bioinformatic prediction). The fundamental unanswered question is whether these interactions occur simultaneously in the same cell population or are mutually exclusive in different HCC subtypes. This apparent complexity underscores the necessity to move beyond descriptive interaction mapping.

### 3.4 Future perspectives

Resolving this complexity demands innovative approaches. Future research must employ single-cell dual-omics (simultaneously measuring lncRNA and miRNA expression) and spatial transcriptomics to determine the co-localization and cell-type specificity of these ZFAS1-miRNA axes within the tumor microenvironment. Furthermore, CRISPR-based genetic screens can systematically identify which ZFAS1-mediated interactions are essential for tumor growth in specific genetic backgrounds. Finally, the therapeutic potential of targeting the ZFAS1 node—through antisense oligonucleotides (ASOs) or small molecules that disrupt its secondary structure—warrants aggressive exploration, as it offers a strategy to simultaneously dampen multiple oncogenic pathways.

## 4 ZFAS1 synergistically drives the molecular mechanisms of HCC through multiple signaling pathways

ZFAS1's regulatory dominance in HCC extends to PI3K/AKT pathway activation, as demonstrated by Luo et al. [56] who identified ATIC-mediated pathway potentiation driving proliferative and migratory capacities. Single-cell RNA sequencing of sorafenib-resistant hepatocellular carcinoma (*Huh7-R*) cells identified pronounced activation of core Notch signaling components, characterized by elevated mRNA levels of Notch receptors (*NOTCH1*-*3*), ligands (*DLL1*, *DLL4*, *JAG1*), and downstream effectors (*HES1*, *HEY1*), concomitant with suppression of pathway inhibitors (*DLL3*, *RBPJ*). Functional validation revealed that ZFAS1 ablation in Huh7 cells significantly attenuates Notch signaling activity, as evidenced by coordinated downregulation of NOTCH1-3 receptors, Delta-like ligands (DLL1-4), and transcriptional targets (HES1/HEY1). These results mechanistically establish ZFAS1 as a critical upstream regulator of Notch-mediated HCC stemness maintenance and EMT progression [57].

While ZFAS1's pathway crosstalk in HCC remains partially characterized, its pan-cancer regulatory versatility reveals therapeutic promise. In triple-negative breast cancer (TNBC), ZFAS1 downregulation correlates with suppressed proliferation/EMT through STAT3 phosphorylation inhibition, whereas it’s silencing paradoxically activates STAT3-driven tumorigenesis [58]. Clear cell renal cell carcinoma (ccRCC) studies unveil ZFAS1-mediated progression via the miR-10a/SKA1 axis, suggesting tissue-specific targeting potential [59]. Pancreatic ductal adenocarcinoma (PDAC) models demonstrate ZFAS1's miR-3924 sequestration activates RHOA/ROCK signaling with concurrent FAK downregulation, accelerating metastasis [60]. Gastric cancer mechanisms involve ZFAS1-NKD2-Wnt/β-catenin axis activation that sustains proliferation, EMT, and chemoresistance, reversible by β-catenin overexpression [61]. Colorectal cancer research confirms ZFAS1's dual regulation of cell cycle (CDK1-mediated G1/S transition) and apoptosis (miR-590–3p sponging→p53 suppression→PARP cleavage inhibition), cementing its genomic stability control [62]. The Notch signaling pathway exhibits extensive cross-talk with oncogenic cascades during tumorigenesis, with particular significance in EMT regulation during cancer progression [63]. Mechanistically, the seminal work by Gao et al. [64] demonstrated that ZFAS1 knockdown modulates Notch pathway components, notably downregulating HES family basic helix-loop-helix transcription factor 1 (HES-1) and reducing nuclear accumulation of the Notch intracellular domain (*NICD*). These findings establish ZFAS1 as a master regulator of Notch signaling activation, positioning it upstream of this critical oncogenic pathway.

Although derived from non-HCC models, these findings collectively establish ZFAS1's evolutionary conservation in coordinating malignancy through core mechanisms (miRNA sequestration/pathway networking), providing trans-cancer insights for deciphering its HCC regulatory architecture.

## 5 ZFAS1 remodels the immunometabolic regulatory role of the HCC tumor microenvironment

The tumor microenvironment (TME) exerts pivotal roles in the initiation, progression, and therapeutic resistance of HCC [65]. Within the TME, immune cells serve as critical orchestrators, engaging in dynamic crosstalk with neoplastic cells to modulate tumor behavior. Emerging evidence indicates that immune cell functionality is intricately linked to cellular metabolism, and metabolic reprogramming of these cells significantly drives HCC advancement by altering cytokine profiles and immunosuppressive signaling [66]. Furthermore, HCC cells undergo intrinsic metabolic adaptations—such as enhanced glycolysis and glutaminolysis—that not only sustain their proliferation and survival but also reshape the TME through aberrant secretion of oncometabolites (e.g. lactate, ketone bodies) and exosomes. These secreted factors establish a nutrient-depleted, acidic niche that promotes stromal activation, immune evasion, and metastatic dissemination [67].

ZFAS1 orchestrates multidimensional remodeling of the TME through immunometabolic reprogramming. In angiogenesis regulation, colorectal cancer studies demonstrate ZFAS1-mediated VEGFA activation via miR-150–5p sequestration, promoting AKT/mTOR-dependent vascularization [68]. This mechanism aligns with HCC characteristics where VEGFA expression positively correlates with microvascular density [69], suggesting conserved angiogenic regulation. Exosomal transfer mechanisms reveal ZFAS1's capacity to activate ERK signaling in gastric cancer cells, driving EMT and metastatic niche formation [70]. Esophageal squamous cell carcinoma models further show exosomal ZFAS1 enhances invasiveness through miR-124/STAT3 axis manipulation, establishing paracrine-mediated TME rewiring [71]. Epigenetically, m6A-modified ZFAS1 strengthens miR-647 binding in cervical cancer, potentiating metastatic spread through miRNA-mediated intercellular crosstalk [72]. While direct HCC evidence remains limited, immune modulation studies suggest potential mechanisms: Exosomal miR-21–5p induces M2-polarized TAMs via SP1/XBP1 [73], and other HCC-associated lncRNAs regulate TAM phenotypes through transcriptional networks [[74], [75], [76], [77], [78]], implicating ZFAS1 in similar immune evasion pathways.

Collectively, ZFAS1 emerges as a molecular linchpin connecting angiogenic and immunosuppressive TME remodeling, though systematic dissection of its HCC-specific interactome (protein complexes/exosomal targeting) requires multi-omics integration with CRISPR-based spatial modeling.

## 6 The translational prospects of ZFAS1 as a liquid biomarker for non-invasive diagnosis and prognostic assessment of HCC

HCC remains one of the most aggressive malignancies with high mortality rates, underscoring the urgent need for early diagnostic tools and innovative therapies, particularly for high-risk populations. LncRNAs have emerged as promising biomarkers due to two key attributes: (1) their high cell- and tissue-specific expression patterns, which may facilitate tumor subclassification and therapeutic response prediction [79]; and (2) their remarkable stability and detectability in bodily fluids, enabling non-invasive clinical applications [80].

ZFAS1 has emerged as a promising liquid biopsy biomarker for HCC, demonstrating significant translational potential. Luo et al. [81] established through multi-phase cohorts that plasma ZFAS1 levels in HCC patients significantly exceed those in healthy controls (P < 0.001) and cirrhotic individuals (P < 0.001), showing positive correlation with AFP levels (P = 0.006). Notably, plasma ZFAS1 alone achieved diagnostic AUC = 0.801 (95 % CI: 0.724–0.875), which synergistically improved to AUC = 0.891 when combined with AFP testing.

Taleb et al. [82] specifically demonstrated elevated circulating ZFAS1 in HCV-related HCC across BCLC stages B (HR = 1.35, P = 0.031) and D (HR = 1.31, P = 0.049) versus cirrhosis, correlating with Child-Pugh grade (Spearman's ρ = 0.267, P = 0.039). However, its limited cirrhosis-HCC differentiation capacity (AUC = 0.70 vs AFP's AUC = 1.00) underscores HCV-specific microenvironmental influences on ZFAS1 regulation. Concurrent mechanistic investigations elucidate ZFAS1's miR-150-mediated ZEB1/MMPs pathway regulation, providing biological rationale for its biomarker utility. Future directions necessitate large-scale multicenter validation across etiologies (HBV/HCV) and early-stage HCC, coupled with integrative strategies combining radiomics and exosomal biomarkers to optimize non-invasive diagnostic precision.

## 7 Molecular mechanisms of ZFAS1-Mediated targeted drug resistance in HCC and reversal strategies

Systemic therapies for HCC have undergone transformative advancements through breakthroughs in targeted and immunotherapeutic approaches, significantly improving outcomes for advanced-stage patients. In 2007, the multi-kinase inhibitor sorafenib—targeting VEGFR, PDGFR, and RAF pathways—demonstrated a median overall survival (mOS) of 10.7 months in advanced HCC (SHARP trial), marking the dawn of targeted therapy [83]. Subsequent iterations of targeted agents further extended survival benefits: regorafenib (RESORCE Phase III trial) achieved an mOS of 10.6 months in sorafenib-refractory patients [84], while cabozantinib (CELESTIAL study) yielded an mOS of 10.2 months in progressive HCC [85].

The advent of immunotherapy introduced PD-1 inhibitors such as nivolumab, which showed a 15 % objective response rate (ORR) as monotherapy (CheckMate 040 trial) [86]. Combination strategies with CTLA-4 inhibitors, including ipilimumab (CheckMate 9DW preliminary data), exhibited promising survival trends in first-line treatment of unresectable HCC (uHCC) [87]. Another PD-1 inhibitor, pembrolizumab (KEYNOTE-224 Phase II trial), demonstrated a 17 % ORR in sorafenib-pretreated patients, reinforcing the potential of immune monotherapy [88]. Notably, synergistic targeted-immunotherapy regimens, such as atezolizumab plus bevacizumab (IMbrave150 trial), achieved a landmark mOS of 19.2 months with a 41 % reduction in disease progression risk [89]. These milestones collectively redefine the therapeutic landscape for advanced HCC, transforming long-term survival into a clinically attainable objective.

ZFAS1 has emerged as a central mediator of therapeutic resistance in HCC, orchestrating multidrug resistance (MDR) through multidimensional molecular networks. Table 3 provides a structured overview of ZFAS1-driven resistance paradigms, mapping molecular effectors to clinically actionable reversal strategies for targeted therapeutics.

**Table 3:** Therapeutic resistance mechanisms mediated by ZFAS1.

Resistance Type	Molecular Mechanism	Key Effectors	Reversal Strategy	Clinical Correlation
Sorafenib resistance	PERK/ATF4-dependent ZFAS1↑ → ER stress adaptation	PERK/ATF4 axis	Pharmacological PERK inhibition	Non-responders: ZFAS1↑ (P < 0.01) [90]
Sorafenib resistance	Cancer stemness maintenance → EMT activation	CD133/EPCAM↓, VIM/ZEB1↓	ZFAS1 ablation (IC50↓62 %)	Tumor sphere formation↓68 % [57]
Donafenib resistance	CREB3→ZFAS1→LSD1/CoREST→p65 activation	CREB3/LSD1/NF-κB	ZFAS1/CREB3 knockdown	ZFAS1-CREB3-p65 co-upregulation (P < 0.01) [91]

**Fig. 3:**
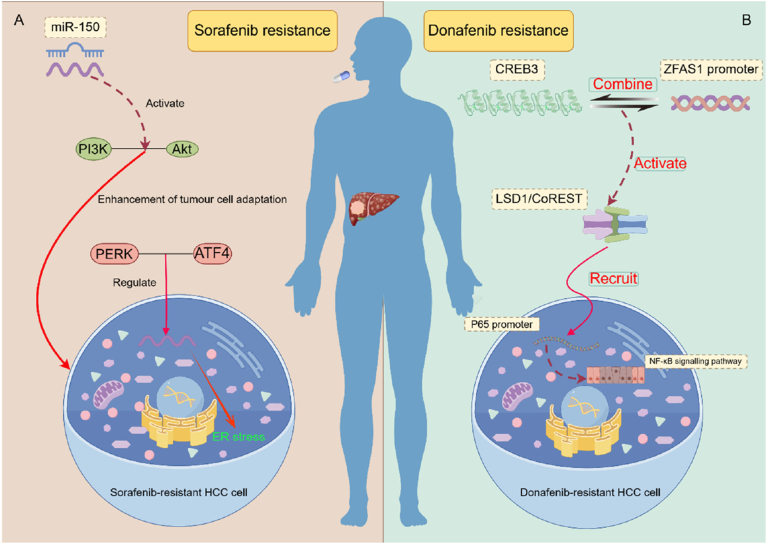
Dual Regulatory Mechanisms of ZFAS1 in Hepatocellular Carcinoma Targeted Therapy Resistance: PERK/ATF4 Axis (sorafenib) and CREB3/p65/NF-κB Pathway (donafenib). 3A: ZFAS1 drives HCC targeted therapy resistance via ER stress by PERK/ATF4-mediated upregulation, which enhances tumor cell adaptability through miR-150 sponging and PI3K/AKT activation, while PERK/ATF4 inhibition restores sorafenib sensitivity. 3B: CREB3 transcriptionally activates ZFAS1, which recruits LSD1/CoREST to the p65 promoter to reduce H3K4 methylation and enhance p65 expression, activating NF-κB-driven proliferation and apoptosis suppression that mediates donafenib resistance; ZFAS1/CREB3 knockdown reduces IC50 (P < 0.001), reversed by p65 overexpression. Created with Figdraw.

Mechanistically, ZFAS1 drives sorafenib resistance via the PERK/ATF4 axis-mediated endoplasmic reticulum (ER) stress adaptation. In resistant HCC models, PERK/ATF4-dependent ZFAS1 upregulation correlates with ER stress activation, while pharmacological PERK inhibition restores drug sensitivity by suppressing ZFAS1 expression [90] (Fig. 3A). Clinical validation confirms ZFAS1's prognostic value in sorafenib non-responders (P < 0.01), with functional studies suggesting dual resistance mechanisms through miR-150 sequestration and PI3K/AKT pathway activation.

ZFAS1 demonstrates marked overexpression in sorafenib-resistant HCC cells, correlating with aggressive clinicopathological features and unfavorable patient outcomes. Genetic ablation of ZFAS1 profoundly compromised clonogenic potential, evidenced by 68 % reduction in tumor sphere formation capacity, concomitant with downregulation of stemness-associated surface markers (*CD133*, *EPCAM*) and activation of caspase-3-dependent apoptotic pathways. Notably, ZFAS1 depletion synergistically enhanced sorafenib efficacy, reducing the half-maximal inhibitory concentration (IC50) by 62 % and increasing cytotoxic response 3.2-fold. Mechanistic interrogation revealed ZFAS1 sustains chemoresistance through tripartite regulation: (1) maintenance of cancer stem cell properties, (2) suppression of mitochondrial apoptosis, and (3) transcriptional control of EMT effectors (*VIM*, *ZEB1*). These findings collectively nominate ZFAS1 as a therapeutically actionable master regulator of sorafenib resistance in HCC, presenting a promising RNA-targeted strategy to circumvent therapeutic failure [57].

Parallel research reveals ZFAS1's role in donafenib resistance via CREB3-mediated transcriptional activation. CREB3 binds the ZFAS1 promoter, inducing LSD1/CoREST complex recruitment to the *p65* promoter (RELA), which enhances NF-κB signaling through H3K4me2 demethylation [91] (Fig. 3B). This epigenetic remodeling reduces apoptosis and increases proliferation, evidenced by IC50 reduction (P < 0.001) upon ZFAS1/CREB3 knockdown and *p65* overexpression-mediated rescue. Clinical correlation analyses demonstrate synchronized upregulation of ZFAS1, CREB3, and p65 in resistant specimens (P < 0.01). These findings establish the CREB3/ZFAS1/NF-κB axis as a promising target for overcoming donafenib resistance.

While the role of ZFAS1 in mediating resistance to targeted therapies in HCC has been preliminarily established, its mechanistic involvement in immune checkpoint inhibitor (ICI) resistance remains unexplored. To date, no studies have elucidated whether ZFAS1 modulates tumor microenvironmental dynamics—such as PD-L1 expression or T-cell infiltration—or orchestrates epigenetic reprogramming (e.g. DNA methylation, chromatin accessibility) to confer resistance to PD-1/PD-L1 inhibitors [[92], [93], [94]]. Notably, the widespread clinical adoption of targeted-immunotherapy combinations (e.g. VEGFR inhibitors with ICIs) in advanced HCC raises a critical unanswered question: Does ZFAS1 drive cross-therapeutic resistance via crosstalk with signaling networks such as the *PI3K-AKT-mTOR* or *JAK-STAT* pathways [95,96]? Addressing this knowledge gap requires systematic investigation. Future studies should employ advanced models (e.g. patient-derived organoids) and cutting-edge technologies (single-cell sequencing, spatial transcriptomics) to delineate the dynamic regulatory networks through which ZFAS1 governs resistance to both mono-immunotherapy and combination regimens. Such efforts may unveil novel therapeutic targets to overcome treatment resistance in HCC.

## 8 Conclusion and perspectives

Despite significant advances in understanding ZFAS1's role in HCC pathogenesis, critical challenges persist regarding its functional heterogeneity, translational barriers, and fragmented mechanistic insights. Furthermore, the functional duality of ZFAS1—exhibiting both oncogenic and tumor-suppressive roles—demands careful contextual interpretation. As highlighted in Section 3.4, this paradox may originate from technical detection variances, microenvironmental reprogramming, or viral etiology. Future multicenter studies should employ single-cell spatial transcriptomics to dissect these context-dependent mechanisms, ultimately enabling precision targeting of ZFAS1 in defined HCC subtypes.

First, ZFAS1's dynamic expression patterns across HCC subtypes (HBV/HCV vs. non-viral) and disease stages remain uncharacterized, while its spatiotemporal regulation within the TME—particularly immunometabolic crosstalk and exosomal communication—requires subtype-specific investigation. Second, current clinical evidence predominantly derives from single-center cohorts, and preclinical models inadequately recapitulate human HCC heterogeneity, constraining ZFAS1's diagnostic/therapeutic translation. Concurrently, liquid biopsy platforms for circulating ZFAS1 detection (especially exosomal) demand improved analytic performance through epigenetic profiling and third-generation sequencing integration.

Although multi-omics approaches have unveiled ZFAS1 interaction networks [55,[97], [98], [99], [100]], its multidimensional roles in cancer stemness, immune evasion, and MDR necessitate systematic validation using spatial transcriptomics, single-cell analytics, and patient-derived organoids. To overcome these limitations, future research should establish interdisciplinary frameworks combining longitudinal clinical cohorts, dynamic omics monitoring, and AI-powered network modeling to comprehensively elucidate ZFAS1's regulatory architecture and microenvironmental dependencies. Such integration will catalyze the transition from mechanistic discovery to personalized theranostics, ultimately propelling HCC precision medicine into a new paradigm.

## CRediT authorship contribution statement

**Peng Zhu:** Writing – original draft, Visualization, Conceptualization. **Hui-ying Liu:** Writing – review & editing, Supervision, Project administration.

## Declaration of generative AI and AI-assisted technologies in the writing process

During the preparation of this work the authors used DeepSeek in order to improve the English language and polish the manuscript. After using this tool/service, the authors reviewed and edited the content as needed and take full responsibility for the content of the published article.

## Declaration of competing interest

The authors declare that they have no known competing financial interests or personal relationships that could have appeared to influence the work reported in this paper.
